# Changes in total and segmental bioelectrical resistance are correlated with whole-body and segmental changes in lean soft tissue following a resistance training intervention

**DOI:** 10.1186/s12970-019-0325-4

**Published:** 2019-11-29

**Authors:** Grant M. Tinsley, Patrick S. Harty, M. Lane Moore, Jozo Grgic, Analiza M. Silva, Luis B. Sardinha

**Affiliations:** 1 Energy Balance & Body Composition Laboratory, Department of Kinesiology & Sport Management, Texas Tech University, Lubbock, TX 79424 USA; 2 Institute for Health and Sport (IHES), Victoria University, Melbourne, Australia; 3 Department of Sport and Health, University of Lisbon, Lisbon, Portugal

**Keywords:** Resistance, Reactance, Phase angle, Bioelectrical impedance analysis, Resistance training, Resistance exercise

## Abstract

**Background:**

Raw bioelectrical values can be used to assess physiological outcomes, though limited information is available concerning the relationships between changes in these values and changes in other variables of interest.

**Methods:**

This investigation quantified the relationships between total and segmental changes in raw bioelectrical variables (i.e., resistance, reactance, and phase angle) and corresponding whole-body and segmental changes in independently assessed body composition. Resistance-trained females (*n* = 31, body mass index: 22.8 ± 2.6 kg/m^2^, body fat: 28 ± 6%) completed eight weeks of supervised resistance training. Before and after the intervention, body composition was assessed via dual-energy x-ray absorptiometry (GE® Lunar Prodigy), and raw bioelectrical variables were assessed via 8-point multi-frequency bioelectrical impedance analysis (Seca® mBCA 515/514) at 19 frequencies ranging from 1 to 1000 kHz.

**Results:**

Lean soft tissue of the whole body (+ 3.2% [2.1, 4.4]; mean [95% confidence interval]) and each body segment (+ 2.8 to 6.3%) increased as a result of the intervention. Group-level changes in total (− 2.4% [− 5.2, 0.3]) and segmental fat mass were not statistically significant. Significant decreases in total resistance (− 2.1% [− 3.7, − 0.6] at 50 kHz) and increases in phase angle (+ 4.2% [2.5, 5.9] at 50 kHz) were observed, with minimal changes in reactance and varying changes in segmental values. Moderate to strong negative correlations (0.63 ≤ |*r*| ≤ 0.83, *p* ≤ 0.001) were found between changes in lean soft tissue and changes in resistance for the whole body, trunk, and arms. No significant correlations were identified between changes in fat mass or bone mineral content and changes in any bioelectrical variable.

**Conclusions:**

Total and segmental changes in resistance were associated with corresponding total and segmental changes in lean soft tissue following a resistance training intervention, while fewer associations were identified between changes in other bioelectrical parameters (i.e., reactance and phase angle) and body composition variables (e.g., fat mass and bone mineral content). Measurement frequency and body segment appeared to influence the presence and strength relationships between bioelectrical and body composition variables. These findings suggest that researchers and practitioners utilizing bioimpedance technology may benefit from examining raw resistance values to enhance detection of physiological adaptations to exercise interventions.

## Introduction

Bioimpedance devices are commonly used to estimate body composition and fluid status due to their low cost, portability, and ease of use [1, 2]. These devices administer electrical currents via surface electrodes and assess the responses of body tissues, producing measures of raw bioelectrical parameters such as resistance (R) and reactance (Xc) [3]. R represents the opposition to the flow of electrical current through body tissues, while Xc is a measure of the delay in conduction caused by cell membranes and other tissue interfaces [4]. A third common parameter, phase angle (φ), is a function of the relationship between R and Xc and has been suggested to serve as a marker of cellular health and integrity [3, 4]. These raw bioelectrical parameters are often utilized to indirectly estimate body fluids or composition using prediction algorithms and assumed coefficients derived from reference data [2]. However, the validity of these estimates has been criticized due to the assumptions and errors associated with the prediction of body components from raw bioelectrical data [5]. Due to these limitations, there is continued interest in utilizing raw bioelectrical parameters, rather than estimates of body fluids or composition predicted by these values, to evaluate physiological outcomes [4].

To date, a variety of investigations have supported the prognostic utility of raw bioelectrical parameters in patients with Human Immunodeficiency Virus infection, cancer, conditions requiring hemodialysis, malnutrition, and anorexia nervosa, suggesting that these measures may be useful for clinicians [4, 6]. Variables such as φ also appear to differentiate between individuals with high or low levels of muscle mass, as cross-sectional investigations have shown moderate positive correlations between φ and fat-free mass (FFM) as well as significant differences in R, Xc, and φ between well-trained bodybuilders and healthy controls [7, 8]. Furthermore, improvements in raw bioelectrical parameters have been demonstrated to occur in conjunction with resistance exercise interventions in a variety of active and inactive populations [9–15]. Though many investigations report cross-sectional associations of raw bioelectrical parameters with aspects of health, disease, and physical performance, relatively limited information is available concerning the relationship between changes in bioelectrical parameters and changes in other variables of interest in response to an intervention (e.g., participation in an exercise program) or disease process. Indeed, to date, no investigation has directly examined the relationship between region-specific changes in bioelectrical variables measured at multiple frequencies and independently quantified changes in region-specific body composition parameters. While many body composition assessment methods provide estimates of whole-body fat and lean mass, fewer provide regional estimates. Of the existing methods used to quantify regional body composition, dual-energy x-ray absorptiometry (DXA) is often recommended and utilized due to its precision and availability [16, 17]. Thus, the purpose of this analysis was to examine the relationship between changes in raw bioelectrical parameters (i.e., R, Xc, and φ) and changes in DXA body composition estimates, for the entire body and specific body regions, in response to a resistance training (RT) intervention.

## Materials and methods

### Overview

The present analysis utilized data collected during a supervised RT intervention in resistance-trained females [18]. Healthy adult females with ≥1 year of RT experience were recruited for participation. At baseline and after eight weeks of supervised RT, assessments via DXA and multi-frequency bioelectrical impedance analysis (MFBIA) were performed. This study was approved by the Texas Tech University Institutional Review Board (IRB2017–912), and all participants provided written informed consent prior to participation. Participants who completed the entire intervention (*n* = 31, age: 22 ± 3 y, height: 165.9 ± 6.6 cm; body mass: 62.8 ± 7.9 kg; body mass index [BMI]: 22.8 ± 2.6 kg/m^2^, body fat: 28 ± 6%) were included in this analysis. These participants were primarily Non-Hispanic Caucasians (*n* = 23), and the remaining participants were Hispanic Caucasians (*n* = 6), Black (*n* = 1), and Asian (n = 1). All participants completed the same RT program and had comparable dietary intakes and physical activity levels throughout the intervention as previously described [18].

### Laboratory visits

Participants reported to the laboratory in athletic clothing after an overnight (≥ 8 h) abstention from eating, drinking, exercising, and consuming caffeine. Metal and accessories were removed, and each participant voided her bladder prior to testing. Urine specific gravity (USG) was assessed via digital refractometer (PA201X-093, Misco, Solon, OH, USA). USG was 1.022 ± 0.005 at the baseline assessment and 1.021 ± 0.005 at the final assessment. After voiding, height was determined via mechanical stadiometer.

### Bioelectrical impedance analysis

An 8-point MFBIA device (mBCA 515/514, Seca® gmbh & co, Hamburg, Germany) with contact electrodes for both hands and both feet was utilized in the present study. This device utilizes 19 frequencies ranging from 1 to 1000 kHz [19]. Previous test-retest reliability assessment in our laboratory with the specific device used in the present investigation produced a SEM of 1.7 Ω (i.e., 0.3%) for R and 0.6 Ω (i.e., 0.9%) for Xc at 50 kHz in a sample of 10 resistance-trained females, with participant repositioning between assessments. In the present investigation, the raw R and Xc values for each device were obtained for all measurement frequencies. These values were used to manually calculate φ (φ = arc tangent [Xc/R] • [180°/π]). In addition to whole-body values, the raw bioelectrical parameters for each body region (i.e. legs, arms, and trunk) were obtained at each frequency.

### Dual-energy X-ray absorptiometry

DXA scans were performed on a Lunar Prodigy scanner (General Electric, Boston, MA, USA) with enCORE software (v. 16.2). The scanner was calibrated using a quality control block each morning prior to use, and positioning of participants was conducted according to manufacturer recommendations. Each participant was able to fit within the scanning dimensions. A trained operator manually adjusted the analysis lines to demarcate body regions (i.e. legs, arms, and torso). In the lower body, all tissue distal to the line placed perpendicular to the femoral neck was designated as the leg region. In the upper body, all tissue distal to the line placed through the glenohumeral joint was designated as the arm region. The trunk region consisted of all tissue inferior to the mandible that was not included in the leg or arm regions. For the whole body and each body region, estimates of lean soft tissue (LST), fat mass (FM), soft tissue (ST; calculated as LST + FM), and bone mineral content (BMC) were obtained. Previous reliability assessment in our laboratory with the specific device used in the present investigation produced SEM values of 0.7% for total LST, 2.1% for total FM, and 0.9% for BMC in a sample of 10 resistance-trained females, although reliability may be higher with the positioning procedures utilized in the present study [20].

### Intervention

All participants completed eight weeks of supervised RT coupled with protein supplementation, and data from all participants completing the intervention were included in the present analysis. Training took place three times per week within the research laboratories under direct researcher supervision. Upper- and lower-body sessions were alternated, with the following exercises included in the overall program at least weekly: barbell deadlift, barbell back squat, hip sled, stiff-leg deadlift, lunges with dumbbells, leg curl machine, leg extension machine, barbell bench press, bent-over dumbbell rows, barbell shoulder press, dumbbell flyes, barbell preacher curls, dumbbell triceps extensions, “skull crushers,” dumbbell curls, and inverted rows (Additional file 1: Table S1). Each session included 5 to 6 of these exercises, with 4 sets of 8 to 12 repetitions completed for most exercises, as previously described [18]. Participants were instructed to train to momentary muscular exhaustion during each set, and the load was adjusted as necessary to ensure compliance with the specified repetition range. Rest intervals between sets and exercises ranged from 90 to 180 s. Following each RT session, participants were provided with 25 g whey protein (Elite 100% Whey, Dymatize Enterprises, LLC, Dallas, TX, USA). Participants were provided with additional whey protein to consume outside the laboratory in order to achieve a daily protein intake of ≥1.4 g/kg [21].

### Statistical analysis

Changes in raw bioelectrical parameters (i.e. ΔR, ΔXc, and Δφ) and DXA variables (i.e. ΔST, ΔLST, ΔFM, and ΔBMC) were expressed as changes between baseline and final values relative to the baseline value (i.e., percent changes), and the associated ranges and 95% confidence intervals were generated. Changes in R, Xc, and φ were quantified for the entire body and each body region at each measurement frequency (i.e. 1, 1.5, 2, 3, 5, 7.5, 10, 15, 20, 30, 50, 75, 100, 150, 200, 300, 500, 750, and 1000 kHz), although results from the standard 50 kHz frequency, along with a representative low frequency (1 kHz) and high frequency (1000 kHz), are presented in the main body of this manuscript. Results from all 19 frequencies are presented in Additional file 2: Table S2, Additional file 3: Table S3 and Additional file 4: Table S4. R and Xc values were not standardized to height (i.e., R/h and Xc/h) or segment length because ΔR and ΔXc are mathematically identical to Δ(R/h) and Δ(Xc/h), assuming no change in height, due to the calculation of changes relative to baseline values in the present analysis. Paired-samples t-tests were used to identify changes in DXA variables and raw bioelectrical parameters across the entire lifestyle intervention. Bonferroni post-hoc adjustments were manually applied to control the familywise error rate within each DXA and bioelectrical variable, yielding a significance level of *p* ≤ 0.003 for DXA variables (0.05/[4 DXA variables • 4 body regions]) and a significance level of *p* ≤ 0.004 for each bioelectrical parameters (0.05/[3 bioelectrical frequencies • 4 body regions]). Pearson correlation coefficients (r) between percent changes in raw bioelectrical parameters and percent changes in DXA variables were calculated for the entire body and each body region. For example, ΔR, ΔXc, and Δφ for the leg region were correlated with DXA ΔST, ΔLST, ΔFM, and ΔLST for the leg region. The accepted statistical significance level for all correlations was adjusted for multiple comparisons using the Bonferroni method for each bioelectrical parameter (i.e. 0.05/[4 DXA variables • 4 body regions • 3 bioelectrical frequencies]), yielding a significance level of *p* ≤ 0.001. Correlations with *p*-values below this threshold were considered statistically significant, and correlations with *p*-values > 0.001 and <  0.05 (i.e. those that would have been statistically significant in the absence of correction for multiple comparisons) were noted as correlations potentially worthy of further exploration. All correlation coefficients were classified as weak (|*r*| ≤ 0.35), moderate (0.36 ≤ |*r*| ≤ 0.67), or strong (0.68 ≤ |*r*| ≤ 1.0) [22]. Data were analyzed using IBM SPSS (v. 25).

## Results

Pre-testing and post-testing values for raw bioelectrical and DXA variables, as well as mean percent changes and the associated ranges and confidence intervals, are displayed in Table 1. Significant (*p* <  0.001) increases in total and segmental LST in all body segments were observed across the eight-week RT intervention. Segmental values for ST increased in the arms only (*p* <  0.0001). No statistically significant group-level changes in FM were detected, although the majority of the 95% confidence intervals were negative, indicative of fat loss, for total, trunk, and leg FM (p: 0.05 to 0.12). Similarly, no group-level changes in BMC were detected. Significant decreases in total R and increases in whole-body φ were detected at the 50 kHz frequency, with no changes in total Xc. Decreases in arm R, an increase in trunk Xc, and increases in trunk and arm φ were also observed at the 50 kHz frequency. At the 1 kHz frequency, only a significant increase in arm φ was detected. At the 1000 kHz frequency, total and arm R, as well as arm Xc, decreased.

**Table 1:** Changes in Body Composition and Raw Bioelectrical Parameters

			Pre	Post	p	Δ% (95% CI)	Range (Δ%)
*DXA*							
	ST (kg)	Total	60.4 ± 7.7	61.3 ± 7.5	0.006	1.5 (0.6, 2.5)	− 5.7 to 5.4
		Trunk	28.4 ± 3.9	28.6 ± 3.7	0.27	0.9 (−0.4, 2.2)	−9.7 to 8.5
		Legs	21.6 ± 3.1	22.0 ± 3.1	0.006	1.8 (0.6, 3.0)	−5.9 to 7.7
		Arms	6.6 ± 1.0	6.9 ± 1.0	< 0.0001^1^	4.3 (2.9, 5.8)	−3.7 to 11.3
	LST (kg)	Total	41.8 ± 4.3	43.1 ± 4.1	< 0.0001^1^	3.2 (2.1, 4.4)	− 3.1 to 10.2
		Trunk	19.9 ± 2.1	20.5 ± 2.0	0.001^1^	2.8 (1.4, 4.3)	−5.1 to 11.5
		Legs	14.4 ± 1.7	14.8 ± 1.7	0.0001^1^	3.5 (2.0, 5.1)	−3.7 to 13.8
		Arms	4.6 ± 0.7	4.8 ± 0.7	< 0.0001^1^	6.3 (4.5, 8.1)	−3.0 to 16.9
	FM (kg)	Total	18.6 ± 5.4	18.2 ± 5.3	0.06	− 2.4 (− 5.2, 0.3)	− 22.8 to 15.4
		Trunk	8.5 ± 3.0	8.2 ± 2.9	0.05	−3.8 (−8.1, 0.5)	− 35.7 to 23.5
		Legs	7.2 ± 2.0	7.1 ± 2.1	0.12	−1.7 (− 3.6, 0.2)	−12.1 to 10.1
		Arms	2.1 ± 0.6	2.1 ± 0.6	0.74	− 0.3 (−3.0, 2.4)	−23.4 to 9.3
	BMC (kg)	Total	2.42 ± 0.23	2.43 ± 0.23	0.34	0.3 (−0.2, 0.8)	−4.7 to 3.1
		Trunk	0.73 ± 0.08	0.73 ± 0.08	0.95	0.1 (−1.3, 1.5)	−10.0 to 10.1
		Legs	0.89 ± 0.12	0.89 ± 0.12	0.74	0.1 (−0.3, 0.5)	−2.5 to 2.4
		Arms	0.30 ± 0.04	0.30 ± 0.04	0.04	1.0 (0.0, 1.9)	−4.7 to 5.4
*MFBIA*							
R (Ω)	1 kHz	Total	806 ± 88	794 ± 75	0.14	−1.2 (−3.1, 0.6)	− 12.2 to 10.9
		Trunk	28 ± 3	28 ± 2	0.80	0.1 (−1.8, 2.0)	− 10.3 to 11.4
		Legs	340 ± 41	339 ± 36	0.84	0.1 (−2.2, 2.4)	−17.6 to 12.7
		Arms	436 ± 50	425 ± 44	0.01	−2.3 (− 4.2, −0.5)	−10.0 to 9.2
	50 kHz	Total	695 ± 80	679 ± 68	0.009^1^	− 2.1 (−3.7, − 0.6)	− 11.2 to 8.3
		Trunk	23 ± 2	23 ± 2	0.15	−0.9 (− 2.4, 0.6)	−9.9 to 6.7
		Legs	289 ± 35	287 ± 31	0.39	−0.6 (− 2.4, 1.3)	−15.5 to 10.1
		Arms	382 ± 47	368 ± 40	0.0004^1^	−3.3 (−4.9, −1.6)	− 10.4 to 6.9
	1000 kHz	Total	593 ± 70	576 ± 60	0.002^1^	− 2.5 (− 4.1, − 1.0)	− 10.9 to 7.5
		Trunk	18 ± 2	18 ± 2	0.01	− 2.4 (− 4.4, − 0.3)	− 15.8 to 10.9
		Legs	243 ± 30	241 ± 26	0.28	−0.8 (− 2.5, 1.0)	−14.6 to 8.7
		Arms	322 ± 40	309 ± 35	0.0001^1^	−3.7 (− 5.2, − 2.2)	− 10.9 to 6.0
Xc (Ω)	1 kHz	Total	16.7 ± 2.4	17.3 ± 2.6	0.20	4.5 (− 0.4, 9.4)	−27.0 to 30.8
		Trunk	2.6 ± 1.3	2.6 ± 0.9	0.90	7.4 (− 4.6, 19.4)	−47.7 to 81.4
		Legs	6.5 ± 1.1	6.8 ± 1.2	0.27	4.4 (−0.8, 9.6)	−31.5 to 32.7
		Arms	7.8 ± 1.0	8.1 ± 1.3	0.10	4.9 (0.2, 9.7)	−19.2 to 33.3
	50 kHz	Total	69.0 ± 8.0	70.2 ± 7.8	0.29	2.1 (−0.9, 5.1)	−17.6 to 19.6
		Trunk	2.8 ± 0.4	2.9 ± 0.4	0.004^1^	6.2 (2.5, 9.9)	−13.4 to 34
		Legs	32.6 ± 4.9	33.1 ± 4.6	0.41	2.2 (−1.3, 5.8)	−24.6 to 22.9
		Arms	36.0 ± 3.8	36.4 ± 3.8	0.48	1.4 (−1.5, 4.4)	−13.3 to 18.8
	1000 kHz	Total	20.4 ± 8.3	20.4 ± 6.2	0.99	9.4 (−3.3, 22)	−32.3 to 128
		Trunk	6.5 ± 2.0	5.5 ± 1.8	0.007	−11.1 (−20.1, − 2.1)	−61.9 to 49.9
		Legs	16.5 ± 4.3	16.3 ± 3.7	0.58	0.3 (−4.2, 4.9)	− 32.7 to 32.5
		Arms	39.2 ± 9.3	36.4 ± 7.9	0.001^1^	−6.1 (−9.4, −2.8)	−20.7 to 18.8
φ (°)	1 kHz	Total	1.2 ± 0.1	1.2 ± 0.2	0.01	5.4 (1.9, 8.9)	−16.9 to 25.9
		Trunk	5.2 ± 2.4	5.2 ± 1.9	0.99	6.8 (−4.4, 17.9)	−45.7 to 72.3
		Legs	1.1 ± 0.1	1.1 ± 0.2	0.06	3.8 (0.5, 7.1)	−16.9 to 22.8
		Arms	1.0 ± 0.1	1.1 ± 0.2	0.002^1^	7.2 (3.3, 11.2)	−12.1 to 41.5
	50 kHz	Total	5.7 ± 0.5	5.9 ± 0.6	< 0.0001^1^	4.2 (2.5, 5.9)	−7.1 to 14.0
		Trunk	6.8 ± 0.7	7.3 ± 1.0	< 0.0001^1^	7.1 (4.0, 10.1)	−5.3 to 29.5
		Legs	6.4 ± 0.6	6.6 ± 0.6	0.02	2.6 (0.7, 4.5)	−10.7 to 12.2
		Arms	5.4 ± 0.4	5.7 ± 0.5	< 0.0001^1^	4.7 (3.1, 6.3)	−5.5 to 15.2
	1000 kHz	Total	1.9 ± 0.6	2.0 ± 0.5	0.33	12.1 (− 0.7, 25)	−26.1 to 127.6
		Trunk	19.6 ± 5.3	17.3 ± 5.1	0.009	−9.2 (−16.9, − 1.6)	− 52.1 to 30.5
		Legs	3.8 ± 0.6	3.8 ± 0.5	0.96	0.7 (− 2.2, 3.7)	− 21.1 to 21.8
		Arms	6.8 ± 0.8	6.6 ± 0.7	0.007	− 2.7 (−4.6, − 0.8)	−12.0 to 12.0

^1^Statistically significant at Bonferroni corrected alpha level of 0.003 (16 comparisons) for DXA variables and Bonferroni corrected alpha level of 0.004 (corrected per frequency, with 12 comparisons per frequency) for MFBIA variables

*Abbreviations*. φ: phase angle, *BMC* bone mineral content, *CI* confidence interval, *DXA* dual-energy x-ray absorptiometry, *FM* fat mass, *LST* lean soft tissue, *MFBIA* multi-frequency bioelectrical impedance analysis, *R* resistance, *ST* soft tissue, *Xc* reactance

**Fig. 1:**
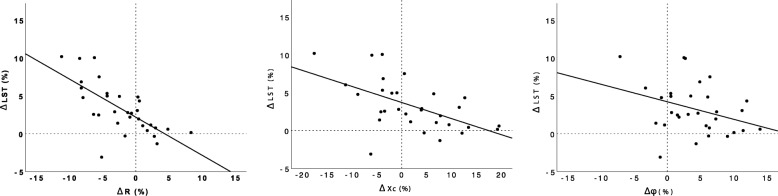
Relationship between whole-body changes in raw bioelectrical variables and changes in total lean soft tissue. Results from 50 kHz frequency are displayed. *Abbreviations:* φ: phase angle assessed by multi-frequency bioelectrical impedance analysis (MFBIA), LST: lean soft tissue assessed by dual-energy x-ray absorptiometry; R: resistance assessed by MFBIA, Xc: phase angle assessed by MFBIA

**Fig. 2:**
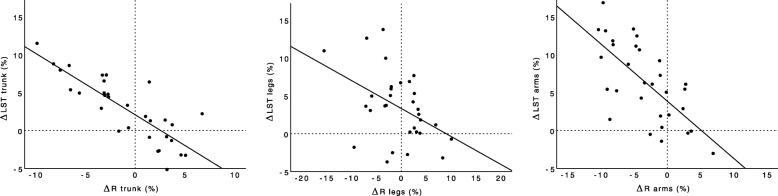
Relationship between segmental changes in resistance and segmental changes in lean soft tissue. Results from 50 kHz frequency are displayed. *Abbreviations:* LST: lean soft tissue assessed by dual-energy x-ray absorptiometry; R: resistance assessed by multi-frequency bioelectrical impedance analysis

Moderate negative correlations (0.56 ≤ |*r*| ≤ 0.62, *p* ≤ 0.001) were detected between changes in both total and segmental ST and the corresponding ΔR values at all three measurement frequencies of interest (Table 2). Similarly, moderate and strong negative correlations (0.63 ≤ |*r*| ≤ 0.83, *p* ≤ 0.001) were found between ΔLST and ΔR for total and segmental changes to the trunk and arms at all three frequencies of interest (Figs. 1 and 2). No significant correlations were identified between ΔFM or ΔBMC and ΔR. Only three significant correlations were observed between ΔXc and DXA variables; segmental arm ΔST exhibited a negative correlation with arm ΔXc at 1000 kHz (*r* = − 0.62), total ΔLST was negatively correlated with ΔXc at 50 kHz (*r* = − 0.56), and arm ΔLST was negatively correlated with ΔXc at 1000 kHz (*r* = − 0.69). No significant correlations were identified between ΔFM or ΔBMC and ΔXc. Correlations between Δφ and DXA variables generally did not reach the threshold of statistical significance, although arm Δφ exhibited a moderate negative correlation with arm ΔLST (*r* = − 0.67) at the 1000 kHz frequency only. Additional file 2: Table S2, Additional file 3: Table S3 and Additional file 4: Table S4 present correlation results for all 19 measurement frequencies.

**Table 2:** Pearson correlations between changes in bioelectrical variables and changes in body composition

		ΔR			ΔXc			Δφ		
		1 kHz	50 kHz	1000 kHz	1 kHz	50 kHz	1000 kHz	1 kHz	50 kHz	1000 kHz
ΔST	Total	− 0.54^2^	− 0.56^1^	− 0.54^2^	− 0.38^2^	− 0.54^2^	0.00	− 0.25	− 0.43^2^	0.07
	Trunk	− 0.61^1^	− 0.62^1^	− 0.50^2^	− 0.13	− 0.33	−0.20	− 0.05	− 0.08	− 0.14
	Legs	−0.35^2^	− 0.38^2^	− 0.37^2^	−0.15	− 0.36^2^	−0.42^2^	0.01	−0.28	− 0.43^2^
	Arms	−0.59^1^	−0.60^1^	− 0.60^1^	−0.45^2^	− 0.53^2^	−0.62^1^	− 0.27	−0.34	− 0.62^2^
ΔLST	Total	−0.63^1^	−0.69^1^	− 0.69^1^	−0.41^2^	− 0.56^1^	−0.04	− 0.23	−0.34	0.06
	Trunk	−0.73^1^	−0.83^1^	− 0.67^1^	−0.17	− 0.46^2^	−0.29	− 0.07	−0.13	− 0.16
	Legs	−0.38^2^	−0.44^2^	− 0.44^2^	−0.16	− 0.34	−0.45^2^	0.02	−0.19	− 0.44^2^
	Arms	−0.65^1^	−0.68^1^	− 0.70^1^	−0.40^2^	− 0.54^2^	−0.69^1^	− 0.18	−0.25	− 0.67^1^
ΔFM	Total	−0.08	−0.04	− 0.01	−0.05	− 0.14	0.06	− 0.04	−0.20	0.05
	Trunk	−0.05	−0.02	− 0.03	0.02	0.06	0.05	0.03	0.07	0.02
	Legs	−0.09	−0.05	− 0.02	−0.07	− 0.17	−0.10	− 0.04	−0.26	− 0.13
	Arms	−0.13	−0.10	− 0.07	−0.22	− 0.19	−0.11	− 0.21	−0.25	− 0.13
ΔBMC	Total	0.15	0.16	0.18	0.03	0.12	−0.02	− 0.03	0.05	− 0.05
	Trunk	0.05	0.06	0.09	0.07	0.13	0.28	0.06	0.11	0.27
	Legs	0.42^2^	0.41^2^	0.42^2^	0.38^2^	0.37^2^	0.39^2^	0.33	0.29	0.37^2^
	Arms	−0.19	−0.20	−0.21	−0.32	−0.19	− 0.26	−0.30	− 0.14	−0.29

All change (Δ) variables were analyzed as percent changes relative to baseline values. ^1^p ≤ 0.0001 (statistically significant after adjustment for multiple comparisons within each bioelectrical variable). ^2^0.0001 < *p* < 0.05

*Abbreviations*. φ: phase angle assessed by multi-frequency bioelectrical impedance analysis (MFBIA), *FM* fat mass, *LST* lean soft tissue, *R* resistance assessed by MFBIA, *ST* soft tissue (i.e. FM + LST), *Xc* reactance assessed by MFBIA

## Discussion

The purpose of the present investigation was to identify the relationships between changes in whole-body and segmental DXA body composition estimates and changes in corresponding total and segmental raw bioelectrical parameters following a RT intervention. To date, this is the first study to directly examine such changes in region-specific DXA parameters and corresponding regional bioelectrical variables measured at many different frequencies. The pattern of decreased R and increased φ following the exercise intervention in the present study is in accordance with the results of similar RT interventions conducted in older women [9, 10, 12–14, 23, 24], young men [11, 24], and young women [11]. Though the physiological mechanisms driving these changes in electrical conductivity are not fully understood, it is likely that increases in LST and decreases in FM resulting from RT alter the cumulative electrical resistance offered by body tissues [4]. Well-hydrated and electrolyte-rich tissues, such as skeletal muscle, are excellent conductors, while adipose tissue is a relatively poor conductor. Consequently, increases in skeletal muscle mass and decreases in adipose tissue mass improve electrical conductivity and thus reduce the overall R of body tissues [4, 12]. Similarly, RT mediated increases in Xc have been theorized to occur due to muscular hypertrophy [12]. Finally, because φ is a function of the relationship between R and Xc (φ = arc tangent [Xc/R] • [180°/π]), the combined effect of increases in Xc and decreases in R act to increase phase angle [5]. Though significant group-level changes in DXA body composition variables were primarily detected for LST estimates in the present investigation, the wide range of changes in FM observed allowed for relationships between the magnitude of individual FM changes and alterations of raw bioelectrical parameters to also be explored.

The findings of this investigation suggest that total and segmental changes in LST resulting from a RT intervention are more consistently correlated with corresponding changes in R rather than changes in Xc and φ in young, resistance-trained females. Significant negative correlations were identified between ΔLST and ΔR at multiple frequencies, suggesting that as participants gained LST, the resistance of their body tissues to electrical current decreased. Slightly weaker correlations were also identified between ΔST and ΔR at the same frequencies, and no significant correlations were found between ΔFM and ΔR. Together, these findings indicate that the relationship between ΔST and ΔR was primarily driven by ΔLST. Fewer significant correlations were identified between ΔXc and ΔST or ΔLST. Finally, only one statistically significant correlation was identified between any Δφ variable and DXA body composition change, specifically between changes in arm φ and ΔLST at the 1000 kHz frequency only. The lack of correlation between changes in body composition and Δφ at the 50 kHz frequency is somewhat contrary to the report of Tomeleri et al. [23], who identified significant correlations between changes in body fat percentage and Δφ (*r* = − 0.58) as well as between changes in DXA-derived skeletal muscle mass and Δφ (*r* = 0.54). However, it is important to note that, in contrast to the college-aged resistance-trained females recruited in the present investigation, the participants recruited by Tomeleri and colleagues [23] were inactive females at least 60 years of age or older. Additionally, the training program in that study generally included more machine-based exercises, somewhat higher repetition ranges (i.e., 10 to 15 repetitions per set) as well as lower training volume per exercise. Thus, it is probable that these divergent results may have been caused by differences in the participant population and exercise training intervention.

The results of the present study have several important implications for researchers and practitioners who employ raw bioelectrical variables to evaluate physiological changes resulting from lifestyle interventions. First, because disparities in the correlations between specific bioelectrical variables and body composition changes were observed, it is recommended that relationships between all available raw bioelectrical parameters (i.e. R, Xc, and φ) and outcome variables of interest be fully explored. In the present investigation, changes in R were more consistently correlated with changes in body composition compared to changes in other bioelectrical parameters. However, the majority of investigations which have examined changes in raw bioelectrical parameters following an exercise training intervention have employed φ as the primary, or only, bioelectrical outcome [10–13, 15, 23, 25]. Therefore, future investigations may benefit from an examination of changes in R and Xc individually to provide a more comprehensive assessment of alterations in bioelectrical variables. An alternative method to evaluate R and Xc is the usage of bioelectrical impedance vector analysis (BIVA), which normalizes R and Xc values to body height and lends itself to graphical interpretation. In the present investigation, the utilization of percent changes in R and Xc relative to baseline values rendered this standardization mathematically unnecessary as ΔR and ΔXc were equivalent to Δ(R/h) and Δ(Xc/h) with the utilized percent change calculation. However, an alternative method of examining R and Xc values to track physiological responses would be to examine changes in R/h and Xc/h without standardization to baseline values. Secondly, this study found that the strength of the relationships between bioelectrical variables and corresponding body composition changes was somewhat affected by measurement frequency, with some relationships emerging at higher frequencies. Because many bioelectrical devices primarily utilize the 50 kHz frequency only [3], it may be advantageous for future studies to employ bioelectrical impedance spectroscopy or MFBIA devices when a more comprehensive picture of changes in bioelectrical parameters is desired. However, the observed between-frequency differences were comparatively minor, supporting the continued utility of the 50 kHz when multiple frequencies are not available. Nonetheless, we show that results obtained with varying frequencies of measurement may not necessarily be uniform, which suggests that researchers should exercise prudence when comparing the results between studies that used different measurement frequencies. Finally, this investigation demonstrated some differing relationships between segmental changes in bioelectrical variables and corresponding body composition changes, suggesting that segmental bioimpedance does indeed provide additional information beyond whole-body measurements and could potentially be useful in evaluating subtle changes in specific tissue segments. However, for traditional usages of bioimpedance, the added complexity of utilizing segmental bioimpedance values may not be justified given the predominantly similar relationships observed for the whole body and specific body regions. Conversely, although the differences observed between total body and regional bioimpedance may be of relatively minimal consequence for basic bioimpedance assessments, the evaluation of segmental changes in bioelectrical parameters could potentially hold value for settings in which a more comprehensive evaluation of physiological changes is desired, provided that the requisite instrumentation is available.

Several key strengths of the present investigation should be noted. The lifestyle intervention was rigorously controlled. All participants were fully supervised during the RT sessions and were provided with sufficient supplemental protein to support hallmark RT-induced adaptations such as increases in muscle size. The body composition and bioelectrical assessments were well-standardized to reduce confounding factors such as exercise, caffeine, pre-testing dietary intake, and changes in hydration status. However, it is important to note that menstrual phase was not controlled, although the absence of a regular menstrual cycle in 20–30% of participants precluded this control measure [18]. Though menstrual status has been shown to have little influence on DXA-derived body composition variables or measures of total body water derived from MFBIA devices [26], it is possible that menstrual phase may have exerted a small confounding effect on the raw bioelectrical parameters collected by this investigation. Unlike investigations using BIVA or similar procedures, the raw bioelectrical parameters were not standardized to participant height, as the use of percent changes with standardization to baseline values of each participant rendered this unnecessary in the context of the present analysis. As adjustment of segmental values to total height is likely inappropriate due to variation in anthropometric proportions, future work could utilize region-specific bioelectrical parameters that have been standardized to segment length. The results of this investigation may not be generalizable to other bioelectrical devices which use different frequencies or electrode configurations, or to other DXA units which employ different proprietary algorithms and correction factors. Finally, the bioelectrical changes reported in the present investigation were observed in the context of consistent LST accretion, a wide range of changes in FM, and minimal changes in BMC due to the short duration of the intervention. Thus, any generalization of the results of this investigation to other contexts in which different patterns of body composition change are exhibited must be made with caution.

## Conclusions

This investigation identified relationships between changes in raw bioelectrical parameters and changes in body composition resulting from a RT intervention in young, resistance-trained females. The most consistent relationships were identified between changes in LST and changes in R, rather than with other raw bioelectrical parameters such as φ or Xc. These findings suggest that researchers and practitioners utilizing bioimpedance technology may benefit from examining raw R values to enhance detection of physiological adaptations to exercise interventions. In addition, the strength and presence of relationships between raw bioimpedance values and body composition varied to some extent based on measurement frequency and body region, although the results also generally support the continued use of the standard whole-body evaluation of bioelectrical parameters at the 50 kHz frequency. Nonetheless, evaluation of segmental bioimpedance variables can potentially be employed by researchers, clinicians, and practitioners who wish to more comprehensively assess changes in various physiological variables in response to an intervention or biological process.

## Supplementary information

**Additional file 1: Table S1.** Resistance training program

**Additional file 2: Table S2.** Pearson correlations between changes in bioelectrical resistance and changes in dual-energy x-ray absorptiometry body composition

**Additional file 3: Table S3.** Pearson correlations between changes in bioelectrical reactance and changes in dual-energy x-ray absorptiometry body composition

**Additional file 4: Table S4.** Pearson correlations between changes in bioelectrical phase angle and changes in dual-energy x-ray absorptiometry body composition

## Data Availability

The datasets used during the current analysis may be available from the corresponding author on reasonable request.

## Abbreviations

BMC: Bone mineral content
BMI: Body mass index
DXA: Dual-energy x-ray absorptiometry
FFM: Fat-free mass
FM: Fat mass
LST: Lean soft tissue
MFBIA: Multi-frequency bioelectrical impedance analysis
R: Resistance
RT: Resistance training
ST: Soft tissue
USG: Urine specific gravity
Xc: Reactance
Φ: Phase angle
