# Supplementary material for: Changes in total and segmental bioelectrical resistance are correlated with whole-body and segmental changes in lean soft tissue following a resistance training intervention
Source: J Int Soc Sports Nutr. 2019 Nov 29;16:58. doi: 10.1186/s12970-019-0325-4 (PMC6883592; doi:10.1186/s12970-019-0325-4)
Supplement: Supplementary file 1 — Additional file 1: Table S1. Resistance training program [file 12970_2019_325_MOESM1_ESM.pdf]

Supplemental Table 1. Resistance training program[1]

Upper Body A[2]

Exercise	W0 – W4	W4 – W8
Bentover DB rows	4 x 8 – 12, 120 s	5 x 6 – 8, 180 s
DB bench press	4 x 8 – 12, 120 s	
BB shoulder press	4 x 8 – 12, 120 s	
DB flyes	4 x 8 – 12, 90 s	
Preacher curls	4 x 8 – 12, 90 s	
Triceps extension	4 x 8 – 12, 90 s	

Lower Body A

Exercise	W0 – W4	W4 – W8
BB deadlift	4 x 8 – 12, 120 s	5 x 6 – 8, 180 s
Hip sled	4 x 8 – 12, 120 s	5 x 6 – 8, 180 s
Lunges with DB	4 x 8 – 12, 120 s	
Leg curls	4 x 8 – 12, 90 s	
Leg extensions	4 x 8 – 12, 90 s	

Upper Body B

Exercise	W0 – W4	W4 – W8
BB bench press	4 x 8 – 12, 120 s	5 x 6 – 8, 180 s
Bentover DB rows	4 x 8 – 12, 120 s	
DB shoulder press	4 x 8 – 12, 120 s	
DB curls	4 x 8 – 12, 90 s	
Skullcrushers	4 x 8 – 12, 90 s	
Inverted rows (BW)	4 x 8 – 12, 90 s	

Lower Body B

Exercise	W0 – W4	W4 – W8
BB back squat	4 x 8 – 12, 120 s	5 x 6 – 8, 180 s
Stiff-leg deadlift	4 x 8 – 12, 120 s	5 x 6 – 8, 180 s
Lunges with DB	4 x 8 – 12, 120 s	
Leg curls	4 x 8 – 12, 90 s	
Leg extensions	4 x 8 – 12, 90 s	

[1]Exercise prescription shown as: sets x repetition range, rest interval. [2]Participants completed sessions in the following order: Upper Body A, Lower Body A, Upper Body B, Lower Body B. These four sessions were alternated in this order throughout the intervention, with adjustment of load as often as necessary to ensure momentary muscular exhaustion in the specified repetition range. BB: barbell; BW: bodyweight; DB: dumbbell; s: seconds; W0: week 0; W4: week 4; W8: week 8.
