# Supplementary material for: Changes in total and segmental bioelectrical resistance are correlated with whole-body and segmental changes in lean soft tissue following a resistance training intervention
Source: J Int Soc Sports Nutr. 2019 Nov 29;16:58. doi: 10.1186/s12970-019-0325-4 (PMC6883592; doi:10.1186/s12970-019-0325-4)
Supplement: Supplementary file 2 — Additional file 2: Table S2. Pearson correlations between changes in bioelectrical resistance and changes in dual-energy x-ray absorptiometry body composition [file 12970_2019_325_MOESM2_ESM.pdf]

**Supplemental Table 2**. Pearson correlations between changes in bioelectrical resistance and changes in dual-energy x-ray absorptiometry body composition.[1]

| | | | 1 kHz | 1.5 kHz | 2 kHz | 3 kHz | 5 kHz | 7.5 kHz | 10 kHz | 15 kHz | 20 kHz | 30 kHz | 50 kHz | 75 kHz | 100 kHz | 150 kHz | 200 kHz | 300 kHz | 500 kHz | 750 kHz | 1000 kHz |
|---|---|---|---|---|---|---|---|---|---|---|---|---|---|---|---|---|---|---|---|---|---|
| Δ ST | Total | *r* | -0.54 | -0.54 | -0.54 | -0.55 | -0.55 | -0.55 | -0.56 | -0.56 | -0.56 | -0.56 | -0.56 | -0.55 | -0.55 | -0.55 | -0.55 | -0.54 | -0.54 | -0.54 | -0.54 |
| | | *p* | 0.002 | 0.002 | 0.002 | 0.001 | 0.001 | 0.001 | 0.001 | 0.001 | 0.001 | 0.001 | 0.001 | 0.001 | 0.001 | 0.001 | 0.002 | 0.002 | 0.002 | 0.002 | 0.002 |
| | Trunk | *r* | -0.61 | -0.60 | -0.60 | -0.59 | -0.61 | -0.61 | -0.58 | -0.59 | -0.60 | -0.61 | -0.62 | -0.63 | -0.63 | -0.63 | -0.63 | -0.62 | -0.60 | -0.56 | -0.50 |
| | | *p* | 0.0002 | 0.0003 | 0.0004 | 0.0004 | 0.0003 | 0.0002 | 0.0007 | 0.0005 | 0.0003 | 0.0003 | 0.0002 | 0.0002 | 0.0001 | 0.0001 | 0.0002 | 0.0002 | 0.0003 | 0.0012 | 0.0046 |
| | Legs | *r* | -0.35 | -0.35 | -0.35 | -0.36 | -0.36 | -0.36 | -0.37 | -0.38 | -0.38 | -0.38 | -0.38 | -0.38 | -0.38 | -0.38 | -0.38 | -0.37 | -0.37 | -0.37 | -0.37 |
| | | *p* | 0.05 | 0.05 | 0.05 | 0.05 | 0.05 | 0.04 | 0.04 | 0.04 | 0.04 | 0.03 | 0.03 | 0.03 | 0.04 | 0.04 | 0.04 | 0.04 | 0.04 | 0.04 | 0.04 |
| | Arms | *r* | -0.59 | -0.59 | -0.59 | -0.59 | -0.59 | -0.59 | -0.59 | -0.60 | -0.60 | -0.60 | -0.60 | -0.60 | -0.60 | -0.60 | -0.61 | -0.61 | -0.61 | -0.60 | -0.60 |
| | | *p* | 0.0005 | 0.0005 | 0.0005 | 0.0005 | 0.0005 | 0.0005 | 0.0004 | 0.0004 | 0.0004 | 0.0004 | 0.0004 | 0.0004 | 0.0003 | 0.0003 | 0.0003 | 0.0003 | 0.0003 | 0.0003 | 0.0003 |
| Δ LST | Total | *r* | -0.63 | -0.63 | -0.64 | -0.64 | -0.65 | -0.66 | -0.66 | -0.67 | -0.68 | -0.69 | -0.69 | -0.69 | -0.69 | -0.69 | -0.69 | -0.69 | -0.69 | -0.69 | -0.69 |
| | | *p* | 0.0001 | 0.0001 | 0.0001 | 0.0001 | 0.0001 | 0.0001 | 0.0001 | <0.0001 | <0.0001 | <0.0001 | <0.0001 | <0.0001 | <0.0001 | <0.0001 | <0.0001 | <0.0001 | <0.0001 | <0.0001 | <0.0001 |
| | Trunk | *r* | -0.73 | -0.70 | -0.72 | -0.75 | -0.77 | -0.80 | -0.81 | -0.82 | -0.83 | -0.83 | -0.83 | -0.82 | -0.82 | -0.81 | -0.80 | -0.78 | -0.77 | -0.73 | -0.67 |
| | | *p* | <0.0001 | <0.0001 | <0.0001 | <0.0001 | <0.0001 | <0.0001 | <0.0001 | <0.0001 | <0.0001 | <0.0001 | <0.0001 | <0.0001 | <0.0001 | <0.0001 | <0.0001 | <0.0001 | <0.0001 | <0.0001 | <0.0001 |
| | Legs | *r* | -0.38 | -0.38 | -0.39 | -0.39 | -0.39 | -0.40 | -0.41 | -0.42 | -0.42 | -0.43 | -0.44 | -0.44 | -0.44 | -0.44 | -0.44 | -0.44 | -0.44 | -0.44 | -0.44 |
| | | *p* | 0.04 | 0.03 | 0.03 | 0.03 | 0.03 | 0.03 | 0.02 | 0.02 | 0.02 | 0.02 | 0.01 | 0.01 | 0.01 | 0.01 | 0.01 | 0.01 | 0.01 | 0.01 | 0.01 |
| | Arms | *r* | -0.65 | -0.65 | -0.64 | -0.65 | -0.65 | -0.66 | -0.66 | -0.67 | -0.67 | -0.68 | -0.68 | -0.68 | -0.69 | -0.69 | -0.69 | -0.70 | -0.70 | -0.70 | -0.70 |
| | | *p* | 0.0001 | 0.0001 | 0.0001 | 0.0001 | 0.0001 | 0.0001 | 0.0001 | <0.0001 | <0.0001 | <0.0001 | <0.0001 | <0.0001 | <0.0001 | <0.0001 | <0.0001 | <0.0001 | <0.0001 | <0.0001 | <0.0001 |
| Δ FM | Total | *r* | -0.08 | -0.07 | -0.07 | -0.07 | -0.07 | -0.07 | -0.07 | -0.06 | -0.06 | -0.05 | -0.04 | -0.04 | -0.03 | -0.03 | -0.02 | -0.02 | -0.02 | -0.01 | -0.01 |
| | | *p* | 0.68 | 0.69 | 0.71 | 0.70 | 0.71 | 0.73 | 0.71 | 0.74 | 0.77 | 0.80 | 0.83 | 0.85 | 0.86 | 0.89 | 0.90 | 0.91 | 0.93 | 0.95 | 0.97 |
| | Trunk | *r* | -0.05 | -0.06 | -0.04 | -0.01 | -0.01 | -0.01 | 0.03 | 0.02 | 0.00 | 0.00 | -0.02 | -0.04 | -0.05 | -0.06 | -0.06 | -0.06 | -0.06 | -0.05 | -0.03 |
| | | *p* | 0.79 | 0.75 | 0.83 | 0.94 | 0.97 | 0.97 | 0.89 | 0.93 | 0.98 | 0.99 | 0.92 | 0.85 | 0.80 | 0.76 | 0.74 | 0.73 | 0.74 | 0.81 | 0.87 |
| | Legs | *r* | -0.09 | -0.09 | -0.09 | -0.10 | -0.09 | -0.09 | -0.09 | -0.08 | -0.08 | -0.07 | -0.05 | -0.05 | -0.04 | -0.03 | -0.03 | -0.03 | -0.02 | -0.02 | -0.02 |
| | | *p* | 0.62 | 0.62 | 0.61 | 0.61 | 0.61 | 0.63 | 0.64 | 0.67 | 0.68 | 0.72 | 0.77 | 0.81 | 0.83 | 0.86 | 0.87 | 0.89 | 0.90 | 0.90 | 0.91 |
| | Arms | *r* | -0.13 | -0.13 | -0.13 | -0.13 | -0.12 | -0.12 | -0.12 | -0.11 | -0.11 | -0.10 | -0.10 | -0.09 | -0.09 | -0.09 | -0.09 | -0.08 | -0.08 | -0.08 | -0.07 |
| | | *p* | 0.49 | 0.49 | 0.50 | 0.50 | 0.51 | 0.52 | 0.53 | 0.55 | 0.56 | 0.58 | 0.61 | 0.62 | 0.63 | 0.64 | 0.64 | 0.65 | 0.67 | 0.68 | 0.69 |
| Δ BMC | Total | *r* | 0.15 | 0.15 | 0.15 | 0.16 | 0.16 | 0.16 | 0.16 | 0.16 | 0.16 | 0.16 | 0.16 | 0.16 | 0.16 | 0.16 | 0.16 | 0.16 | 0.17 | 0.17 | 0.18 |
| | | *p* | 0.41 | 0.41 | 0.41 | 0.40 | 0.39 | 0.39 | 0.40 | 0.40 | 0.39 | 0.38 | 0.38 | 0.38 | 0.38 | 0.38 | 0.38 | 0.38 | 0.37 | 0.35 | 0.33 |
| | Trunk | *r* | 0.05 | 0.04 | 0.06 | 0.09 | 0.10 | 0.11 | 0.13 | 0.11 | 0.10 | 0.09 | 0.06 | 0.04 | 0.03 | 0.02 | 0.01 | -0.01 | 0.01 | 0.04 | 0.09 |
| | | *p* | 0.78 | 0.81 | 0.73 | 0.62 | 0.59 | 0.56 | 0.50 | 0.55 | 0.59 | 0.63 | 0.74 | 0.85 | 0.88 | 0.92 | 0.95 | 0.94 | 0.95 | 0.83 | 0.63 |
| | Legs | *r* | 0.42 | 0.41 | 0.41 | 0.41 | 0.41 | 0.41 | 0.41 | 0.41 | 0.41 | 0.41 | 0.41 | 0.41 | 0.42 | 0.42 | 0.42 | 0.42 | 0.42 | 0.42 | 0.42 |
| | | *p* | 0.02 | 0.02 | 0.02 | 0.02 | 0.02 | 0.02 | 0.02 | 0.02 | 0.02 | 0.02 | 0.02 | 0.02 | 0.02 | 0.02 | 0.02 | 0.02 | 0.02 | 0.02 | 0.02 |
| | Arms | *r* | -0.19 | -0.19 | -0.18 | -0.19 | -0.19 | -0.19 | -0.20 | -0.20 | -0.20 | -0.20 | -0.20 | -0.20 | -0.21 | -0.21 | -0.21 | -0.21 | -0.21 | -0.21 | -0.21 |
| | | *p* | 0.30 | 0.31 | 0.32 | 0.31 | 0.31 | 0.30 | 0.29 | 0.29 | 0.28 | 0.28 | 0.28 | 0.27 | 0.27 | 0.26 | 0.26 | 0.25 | 0.25 | 0.25 | 0.26 |

[1]Dual-energy x-ray absorptiometry (Lunar Prodigy, GE) variables are displayed in furthest left column, and multi-frequency bioelectrical impedance (mBCA 515/514, Seca) analysis frequencies are displayed in top row.

*Abbreviations.* BMC: bone mineral content; DXA: dual-energy x-ray absorptiometry; FM: fat mass; LST: lean soft tissue; ST: soft tissue (i.e. FM + LST)
