# Supplementary material for: Changes in total and segmental bioelectrical resistance are correlated with whole-body and segmental changes in lean soft tissue following a resistance training intervention
Source: J Int Soc Sports Nutr. 2019 Nov 29;16:58. doi: 10.1186/s12970-019-0325-4 (PMC6883592; doi:10.1186/s12970-019-0325-4)
Supplement: Supplementary file 3 — Additional file 3: Table S3. Pearson correlations between changes in bioelectrical reactance and changes in dual-energy x-ray absorptiometry body composition [file 12970_2019_325_MOESM3_ESM.pdf]

**Supplemental Table 3**. Pearson correlations between changes in bioelectrical reactance and changes in dual-energy x-ray absorptiometry body composition.[1]

| | | | 1 kHz | 1.5 kHz | 2 kHz | 3 kHz | 5 kHz | 7.5 kHz | 10 kHz | 15 kHz | 20 kHz | 30 kHz | 50 kHz | 75 kHz | 100 kHz | 150 kHz | 200 kHz | 300 kHz | 500 kHz | 750 kHz | 1000 kHz |
|---|---|---|---|---|---|---|---|---|---|---|---|---|---|---|---|---|---|---|---|---|---|
| Δ ST | Total | *r* | -0.38 | -0.40 | -0.41 | -0.42 | -0.43 | -0.44 | -0.46 | -0.48 | -0.50 | -0.51 | -0.54 | -0.55 | -0.55 | -0.55 | -0.54 | -0.49 | -0.33 | -0.14 | 0.00 |
| | | *p* | 0.04 | 0.03 | 0.02 | 0.02 | 0.02 | 0.01 | 0.009 | 0.006 | 0.005 | 0.003 | 0.002 | 0.001 | 0.001 | 0.001 | 0.002 | 0.005 | 0.070 | 0.45 | 0.99 |
| | Trunk | *r* | -0.13 | -0.13 | -0.15 | -0.17 | -0.22 | -0.25 | -0.29 | -0.31 | -0.33 | -0.33 | -0.33 | -0.22 | -0.17 | -0.07 | -0.03 | 0.36 | -0.13 | -0.18 | -0.20 |
| | | *p* | 0.48 | 0.48 | 0.43 | 0.37 | 0.23 | 0.18 | 0.11 | 0.09 | 0.07 | 0.07 | 0.07 | 0.23 | 0.36 | 0.71 | 0.87 | 0.05 | 0.50 | 0.34 | 0.28 |
| | Legs | *r* | -0.15 | -0.16 | -0.17 | -0.19 | -0.21 | -0.23 | -0.25 | -0.28 | -0.30 | -0.32 | -0.36 | -0.38 | -0.39 | -0.40 | -0.41 | -0.42 | -0.42 | -0.42 | -0.42 |
| | | *p* | 0.43 | 0.38 | 0.36 | 0.32 | 0.26 | 0.21 | 0.17 | 0.13 | 0.10 | 0.08 | 0.05 | 0.04 | 0.03 | 0.02 | 0.02 | 0.02 | 0.02 | 0.02 | 0.02 |
| | Arms | *r* | -0.45 | -0.46 | -0.47 | -0.48 | -0.49 | -0.50 | -0.51 | -0.52 | -0.52 | -0.53 | -0.53 | -0.54 | -0.54 | -0.55 | -0.56 | -0.58 | -0.61 | -0.62 | -0.62 |
| | | *p* | 0.01 | 0.009 | 0.008 | 0.007 | 0.005 | 0.004 | 0.003 | 0.003 | 0.002 | 0.002 | 0.002 | 0.002 | 0.002 | 0.001 | 0.001 | 0.0006 | 0.0003 | 0.0002 | 0.0002 |
| Δ LST | Total | *r* | -0.41 | -0.38 | -0.36 | -0.35 | -0.36 | -0.39 | -0.42 | -0.46 | -0.48 | -0.52 | -0.56 | -0.58 | -0.59 | -0.58 | -0.56 | -0.50 | -0.32 | -0.13 | -0.04 |
| | | *p* | 0.02 | 0.03 | 0.05 | 0.05 | 0.04 | 0.03 | 0.02 | 0.01 | 0.006 | 0.003 | 0.001 | 0.001 | 0.001 | 0.001 | 0.001 | 0.005 | 0.08 | 0.47 | 0.82 |
| | Trunk | *r* | -0.17 | -0.19 | -0.20 | -0.20 | -0.24 | -0.28 | -0.38 | -0.45 | -0.49 | -0.52 | -0.46 | -0.37 | -0.29 | -0.16 | -0.15 | 0.29 | 0.01 | -0.29 | -0.29 |
| | | *p* | 0.36 | 0.31 | 0.27 | 0.28 | 0.19 | 0.13 | 0.04 | 0.01 | 0.005 | 0.003 | 0.009 | 0.04 | 0.11 | 0.38 | 0.43 | 0.12 | 0.96 | 0.12 | 0.12 |
| | Legs | *r* | -0.16 | -0.16 | -0.16 | -0.17 | -0.19 | -0.21 | -0.23 | -0.25 | -0.27 | -0.30 | -0.34 | -0.36 | -0.38 | -0.40 | -0.41 | -0.42 | -0.44 | -0.45 | -0.45 |
| | | *p* | 0.39 | 0.39 | 0.38 | 0.37 | 0.31 | 0.26 | 0.22 | 0.17 | 0.14 | 0.10 | 0.06 | 0.04 | 0.04 | 0.03 | 0.02 | 0.02 | 0.01 | 0.01 | 0.01 |
| | Arms | *r* | -0.40 | -0.41 | -0.41 | -0.43 | -0.45 | -0.47 | -0.49 | -0.51 | -0.52 | -0.53 | -0.54 | -0.55 | -0.56 | -0.59 | -0.61 | -0.64 | -0.67 | -0.69 | -0.69 |
| | | *p* | 0.03 | 0.02 | 0.02 | 0.02 | 0.01 | 0.007 | 0.005 | 0.004 | 0.003 | 0.002 | 0.002 | 0.001 | 0.001 | 0.0005 | 0.0003 | 0.0001 | <0.0001 | <0.0001 | <0.0001 |
| Δ FM | Total | *r* | -0.05 | -0.10 | -0.13 | -0.15 | -0.16 | -0.17 | -0.16 | -0.16 | -0.15 | -0.14 | -0.14 | -0.13 | -0.13 | -0.13 | -0.14 | -0.13 | -0.09 | -0.03 | 0.06 |
| | | *p* | 0.78 | 0.58 | 0.49 | 0.42 | 0.38 | 0.37 | 0.39 | 0.40 | 0.42 | 0.44 | 0.47 | 0.48 | 0.47 | 0.47 | 0.47 | 0.47 | 0.63 | 0.88 | 0.77 |
| | Trunk | *r* | 0.02 | 0.04 | 0.04 | 0.04 | 0.04 | 0.06 | 0.07 | 0.09 | 0.09 | 0.09 | 0.06 | 0.07 | 0.06 | 0.05 | 0.08 | 0.12 | -0.10 | 0.08 | 0.05 |
| | | *p* | 0.91 | 0.84 | 0.83 | 0.83 | 0.84 | 0.76 | 0.72 | 0.64 | 0.62 | 0.63 | 0.76 | 0.69 | 0.76 | 0.78 | 0.69 | 0.52 | 0.61 | 0.68 | 0.80 |
| | Legs | *r* | -0.07 | -0.09 | -0.10 | -0.13 | -0.14 | -0.15 | -0.16 | -0.16 | -0.16 | -0.17 | -0.17 | -0.17 | -0.17 | -0.16 | -0.16 | -0.14 | -0.12 | -0.11 | -0.10 |
| | | *p* | 0.72 | 0.63 | 0.58 | 0.50 | 0.44 | 0.42 | 0.40 | 0.38 | 0.38 | 0.37 | 0.36 | 0.36 | 0.37 | 0.38 | 0.40 | 0.45 | 0.52 | 0.57 | 0.59 |
| | Arms | *r* | -0.22 | -0.22 | -0.22 | -0.21 | -0.20 | -0.20 | -0.20 | -0.20 | -0.20 | -0.20 | -0.19 | -0.18 | -0.17 | -0.16 | -0.15 | -0.14 | -0.13 | -0.12 | -0.11 |
| | | *p* | 0.23 | 0.23 | 0.24 | 0.26 | 0.27 | 0.28 | 0.28 | 0.28 | 0.28 | 0.28 | 0.30 | 0.34 | 0.36 | 0.40 | 0.42 | 0.45 | 0.47 | 0.51 | 0.56 |
| Δ BMC | Total | *r* | 0.03 | 0.04 | 0.04 | 0.05 | 0.06 | 0.07 | 0.08 | 0.09 | 0.09 | 0.10 | 0.12 | 0.12 | 0.12 | 0.10 | 0.08 | 0.04 | -0.03 | -0.05 | -0.02 |
| | | *p* | 0.85 | 0.84 | 0.82 | 0.80 | 0.76 | 0.72 | 0.68 | 0.65 | 0.61 | 0.57 | 0.53 | 0.53 | 0.53 | 0.60 | 0.68 | 0.85 | 0.89 | 0.80 | 0.91 |
| | Trunk | *r* | 0.07 | 0.05 | 0.06 | 0.06 | 0.07 | 0.10 | 0.17 | 0.19 | 0.19 | 0.21 | 0.13 | 0.12 | 0.09 | 0.08 | 0.18 | -0.06 | -0.01 | 0.33 | 0.28 |
| | | *p* | 0.71 | 0.77 | 0.73 | 0.75 | 0.69 | 0.59 | 0.36 | 0.32 | 0.31 | 0.25 | 0.49 | 0.51 | 0.62 | 0.66 | 0.33 | 0.76 | 0.97 | 0.07 | 0.13 |
| | Legs | *r* | 0.38 | 0.38 | 0.38 | 0.38 | 0.37 | 0.38 | 0.38 | 0.37 | 0.37 | 0.37 | 0.37 | 0.36 | 0.36 | 0.36 | 0.36 | 0.37 | 0.37 | 0.38 | 0.39 |
| | | *p* | 0.03 | 0.03 | 0.03 | 0.04 | 0.04 | 0.04 | 0.04 | 0.04 | 0.04 | 0.04 | 0.04 | 0.04 | 0.04 | 0.05 | 0.04 | 0.04 | 0.04 | 0.03 | 0.03 |
| | Arms | *r* | -0.32 | -0.27 | -0.23 | -0.19 | -0.16 | -0.15 | -0.16 | -0.17 | -0.17 | -0.19 | -0.19 | -0.19 | -0.19 | -0.19 | -0.19 | -0.19 | -0.22 | -0.25 | -0.26 |
| | | *p* | 0.08 | 0.14 | 0.21 | 0.30 | 0.38 | 0.41 | 0.40 | 0.37 | 0.35 | 0.31 | 0.30 | 0.30 | 0.31 | 0.32 | 0.32 | 0.30 | 0.23 | 0.17 | 0.15 |

[1]Dual-energy x-ray absorptiometry (Lunar Prodigy, GE) variables are displayed in furthest left column, and multi-frequency bioelectrical impedance (mBCA 515/514, Seca) analysis frequencies are displayed in top row.

*Abbreviations*. BMC: bone mineral content; DXA: dual-energy x-ray absorptiometry; FM: fat mass; LST: lean soft tissue; ST: soft tissue (i.e. FM + LST)
