# Supplementary material for: Changes in total and segmental bioelectrical resistance are correlated with whole-body and segmental changes in lean soft tissue following a resistance training intervention
Source: J Int Soc Sports Nutr. 2019 Nov 29;16:58. doi: 10.1186/s12970-019-0325-4 (PMC6883592; doi:10.1186/s12970-019-0325-4)
Supplement: Supplementary file 4 — Additional file 4: Table S4. Pearson correlations between changes in bioelectrical phase angle and changes in dual-energy x-ray absorptiometry body composition [file 12970_2019_325_MOESM4_ESM.pdf]

Supplemental Table 4. Pearson correlations between changes in bioelectrical phase angle and changes in dual-energy x-ray absorptiometry body composition.[1]

			1 kHz	1.5 kHz	2 kHz	3 kHz	5 kHz	7.5 kHz	10 kHz	15 kHz	20 kHz	30 kHz	50 kHz	75 kHz	100 kHz	150 kHz	200 kHz	300 kHz	500 kHz	750 kHz	1000 kHz
Δ ST	Total	*r*	-0.25	-0.27	-0.27	-0.28	-0.30	-0.32	-0.35	-0.37	-0.39	-0.41	-0.43	-0.43	-0.42	-0.40	-0.35	-0.26	-0.10	0.00	0.07
		p	0.18	0.15	0.14	0.12	0.10	0.08	0.05	0.04	0.03	0.02	0.02	0.02	0.02	0.03	0.06	0.17	0.58	0.98	0.69
	Trunk	*r*	-0.05	-0.03	-0.04	-0.05	-0.08	-0.09	-0.12	-0.12	-0.12	-0.11	-0.08	0.01	0.02	0.03	-0.01	0.36	-0.11	-0.13	-0.14
		p	0.80	0.87	0.83	0.81	0.65	0.62	0.52	0.53	0.52	0.56	0.66	0.96	0.90	0.86	0.96	0.04	0.57	0.48	0.46
	Legs	*r*	0.01	-0.01	-0.02	-0.05	-0.09	-0.12	-0.14	-0.18	-0.20	-0.23	-0.28	-0.31	-0.34	-0.37	-0.40	-0.42	-0.44	-0.43	-0.43
		p	0.95	0.98	0.91	0.81	0.65	0.52	0.45	0.35	0.29	0.21	0.13	0.09	0.06	0.04	0.03	0.02	0.01	0.01	0.02
	Arms	*r*	-0.27	-0.28	-0.28	-0.29	-0.32	-0.34	-0.35	-0.36	-0.37	-0.36	-0.34	-0.32	-0.32	-0.35	-0.40	-0.47	-0.58	-0.62	-0.62
		p	0.15	0.13	0.13	0.11	0.08	0.06	0.05	0.04	0.04	0.05	0.06	0.08	0.08	0.05	0.03	0.01	0.00	0.00	0.00
Δ LST	Total	*r*	-0.23	-0.18	-0.14	-0.13	-0.14	-0.17	-0.22	-0.26	-0.29	-0.31	-0.34	-0.34	-0.32	-0.28	-0.22	-0.13	-0.02	0.05	0.06
		p	0.21	0.34	0.44	0.50	0.46	0.37	0.23	0.16	0.12	0.09	0.06	0.06	0.08	0.13	0.23	0.50	0.93	0.77	0.76
	Trunk	*r*	-0.07	-0.08	-0.08	-0.04	-0.05	-0.07	-0.13	-0.19	-0.21	-0.22	-0.13	-0.08	-0.04	-0.03	-0.12	0.30	0.04	-0.20	-0.16
		p	0.72	0.68	0.69	0.83	0.78	0.69	0.47	0.31	0.25	0.22	0.49	0.68	0.81	0.86	0.52	0.11	0.83	0.29	0.39
	Legs	*r*	0.02	0.02	0.01	0.00	-0.03	-0.06	-0.08	-0.11	-0.13	-0.16	-0.19	-0.22	-0.24	-0.28	-0.31	-0.36	-0.41	-0.43	-0.44
		p	0.93	0.92	0.95	0.99	0.86	0.74	0.66	0.55	0.48	0.39	0.29	0.23	0.19	0.13	0.09	0.05	0.02	0.02	0.01
	Arms	*r*	-0.18	-0.17	-0.17	-0.18	-0.22	-0.25	-0.27	-0.28	-0.28	-0.27	-0.25	-0.25	-0.25	-0.31	-0.37	-0.48	-0.61	-0.66	-0.67
		p	0.34	0.37	0.37	0.33	0.24	0.17	0.14	0.12	0.12	0.13	0.17	0.18	0.17	0.09	0.04	0.01	0.00	0.00	0.00
Δ FM	Total	*r*	-0.04	-0.11	-0.15	-0.19	-0.21	-0.21	-0.20	-0.20	-0.20	-0.20	-0.20	-0.21	-0.22	-0.22	-0.22	-0.19	-0.10	-0.03	0.05
		p	0.84	0.55	0.42	0.32	0.27	0.25	0.27	0.27	0.27	0.28	0.27	0.26	0.24	0.23	0.24	0.32	0.61	0.89	0.77
	Trunk	*r*	0.03	0.05	0.05	0.05	0.05	0.07	0.07	0.10	0.11	0.11	0.07	0.10	0.07	0.06	0.08	0.12	-0.10	0.06	0.02
		p	0.88	0.78	0.79	0.81	0.81	0.72	0.72	0.60	0.55	0.55	0.70	0.61	0.69	0.74	0.68	0.52	0.59	0.74	0.90
	Legs	*r*	-0.04	-0.08	-0.10	-0.13	-0.16	-0.17	-0.18	-0.20	-0.21	-0.22	-0.26	-0.28	-0.29	-0.30	-0.29	-0.26	-0.20	-0.15	-0.13
		p	0.83	0.68	0.60	0.48	0.39	0.35	0.33	0.29	0.27	0.22	0.17	0.13	0.11	0.10	0.11	0.16	0.28	0.41	0.47
	Arms	*r*	-0.21	-0.23	-0.22	-0.22	-0.22	-0.23	-0.23	-0.24	-0.25	-0.25	-0.25	-0.24	-0.24	-0.22	-0.22	-0.21	-0.19	-0.16	-0.13
		p	0.25	0.22	0.23	0.24	0.23	0.22	0.21	0.20	0.18	0.17	0.17	0.19	0.20	0.23	0.24	0.27	0.32	0.38	0.48
Δ BMC	Total	*r*	-0.03	-0.04	-0.04	-0.03	-0.02	-0.01	0.01	0.02	0.03	0.04	0.05	0.05	0.03	-0.01	-0.05	-0.10	-0.12	-0.10	-0.05
		p	0.85	0.83	0.84	0.87	0.91	0.97	0.96	0.91	0.87	0.83	0.79	0.81	0.86	0.96	0.79	0.61	0.53	0.61	0.81
	Trunk	*r*	0.06	0.05	0.06	0.04	0.06	0.08	0.15	0.17	0.18	0.22	0.11	0.12	0.08	0.08	0.18	-0.06	-0.01	0.32	0.27
		p	0.73	0.79	0.77	0.81	0.76	0.66	0.43	0.35	0.33	0.25	0.54	0.54	0.65	0.68	0.34	0.75	0.97	0.08	0.14
	Legs	*r*	0.33	0.34	0.34	0.34	0.33	0.33	0.33	0.33	0.32	0.31	0.29	0.27	0.26	0.25	0.26	0.28	0.32	0.35	0.37
		p	0.07	0.06	0.06	0.07	0.07	0.07	0.07	0.07	0.08	0.09	0.12	0.14	0.16	0.17	0.16	0.13	0.08	0.05	0.04
	Arms	*r*	-0.30	-0.25	-0.20	-0.15	-0.11	-0.09	-0.09	-0.11	-0.12	-0.13	-0.14	-0.13	-0.12	-0.11	-0.11	-0.14	-0.21	-0.27	-0.29
		p	0.11	0.18	0.28	0.43	0.57	0.62	0.61	0.57	0.53	0.48	0.46	0.47	0.53	0.55	0.54	0.47	0.25	0.14	0.11

[1]Dual-energy x-ray absorptiometry (Lunar Prodigy, GE) variables are displayed in furthest left column, and multi-frequency bioelectrical impedance (mBCA 515/514, Seca) analysis frequencies are displayed in top row.

Abbreviations. BMC: bone mineral content; DXA: dual-energy x-ray absorptiometry; FM: fat mass; LST: lean soft tissue; ST: soft tissue (i.e. FM + LST)
